# Effective Management of Transuretero-Ureterocutaneostomy (TUUC)-Related Stenosis Using a Combined Antegrade and Retrograde Approach With Holmium Laser Endoureterotomy: A Case Report

**DOI:** 10.7759/cureus.86594

**Published:** 2025-06-23

**Authors:** Indrawarman Soerohardjo, Ahmad Zulfan Hendri, Ahmad Shafwan Natsir, Narpati Wesa Pikatan, Toni Febriyanto

**Affiliations:** 1 Urology Division, Faculty of Medicine, Public Health and Nursing, Universitas Gadjah Mada, Yogyakarta, IDN

**Keywords:** holmium laser, minimally invasive surgery, transuretero-ureterocutaneostomy, ureteral stenosis, urinary diversion

## Abstract

Bladder cancer (BC) is the seventh most common cancer globally, with significant morbidity and mortality. Radical cystectomy is the primary treatment for muscle-invasive bladder cancer (MIBC), often followed by urinary diversion techniques such as transuretero-ureterocutaneostomy (TUUC). While TUUC can be an effective alternative to urinary diversions, it is associated with complications, including anastomotic stenosis. A 60-year-old male with BC underwent radical cystectomy followed by TUUC. Postoperatively, the patient developed anastomotic stenosis, initially managed with stent placement. However, left flank pain and oliguria persisted, and imaging revealed grade 2 left hydronephrosis and elevated creatinine levels. Two operators performed the procedure, using antegrade access percutaneously as a guiding approach and retrograde access through the TUUC orifice to perform the holmium laser endoureterotomy. The holmium laser endoureterotomy was successfully performed using a dual-access technique, combining both antegrade and retrograde approaches to enhance precision and minimize tissue damage. Post-procedure, the patient experienced significant relief from symptoms, and renal function improved markedly, with decreased creatinine levels. This case underscores the effectiveness of a combined approach of holmium laser endoureterotomy in managing TUUC-related stenosis. It offers a minimally invasive solution for maintaining patency and enhancing patient outcomes after complex urinary diversion procedures.

## Introduction

Bladder cancer (BC) is the seventh most common cancer globally, with over 1.7 million prevalent cases and significant mortality. In Indonesia, BC ranks 13th in incidence, with 7,381 new cases reported in 2022. Radical cystectomy is the primary treatment for muscle-invasive bladder cancer (MIBC), often combined with urinary diversion techniques such as transuretero-ureterocutaneostomy (TUUC). TUUC avoids complications linked to bowel anastomosis but can lead to anastomotic stenosis [1]. Non-muscle invasive bladder cancer (NMIBC) is treated with transurethral resection of bladder tumor (TURBT), while MIBC typically requires cystectomy. TUUC, first introduced by Higgins in 1935, provides a viable alternative to the ileal conduit (IC) method by minimizing bowel-related complications. Despite its effectiveness for specific indications, TUUC is associated with significant challenges and complications, particularly developing anastomotic strictures [2]. This study aims to share the management of TUUC anastomotic stricture through endoscopic procedures to reduce the risk post-operation.

## Case presentation

A 60-year-old patient with a diagnosis of BC presented with complaints of painful urination accompanied by blood for one year before seeking medical attention in July 2023. Imaging revealed an intra-bladder mass extending to surrounding organs. The patient was scheduled for a radical cystectomy followed by urinary diversion reconstruction via TUUC. The tumor, measuring 11.5 x 11 x 4.5 cm, was removed and sent for histopathological examination, which confirmed infiltrating urothelial carcinoma. Tumor cells were found to invade the prostate, but no lymph node involvement was detected, classifying the cancer as stage T4a. Radiological scans of the thorax showed no metastasis to the lungs or spine. The patient's urine production returned to normal postoperatively, and the pain complaints decreased. The patient was scheduled for monthly follow-ups to receive adjuvant chemotherapy with gemcitabine and carboplatin.

**Figure 1:**
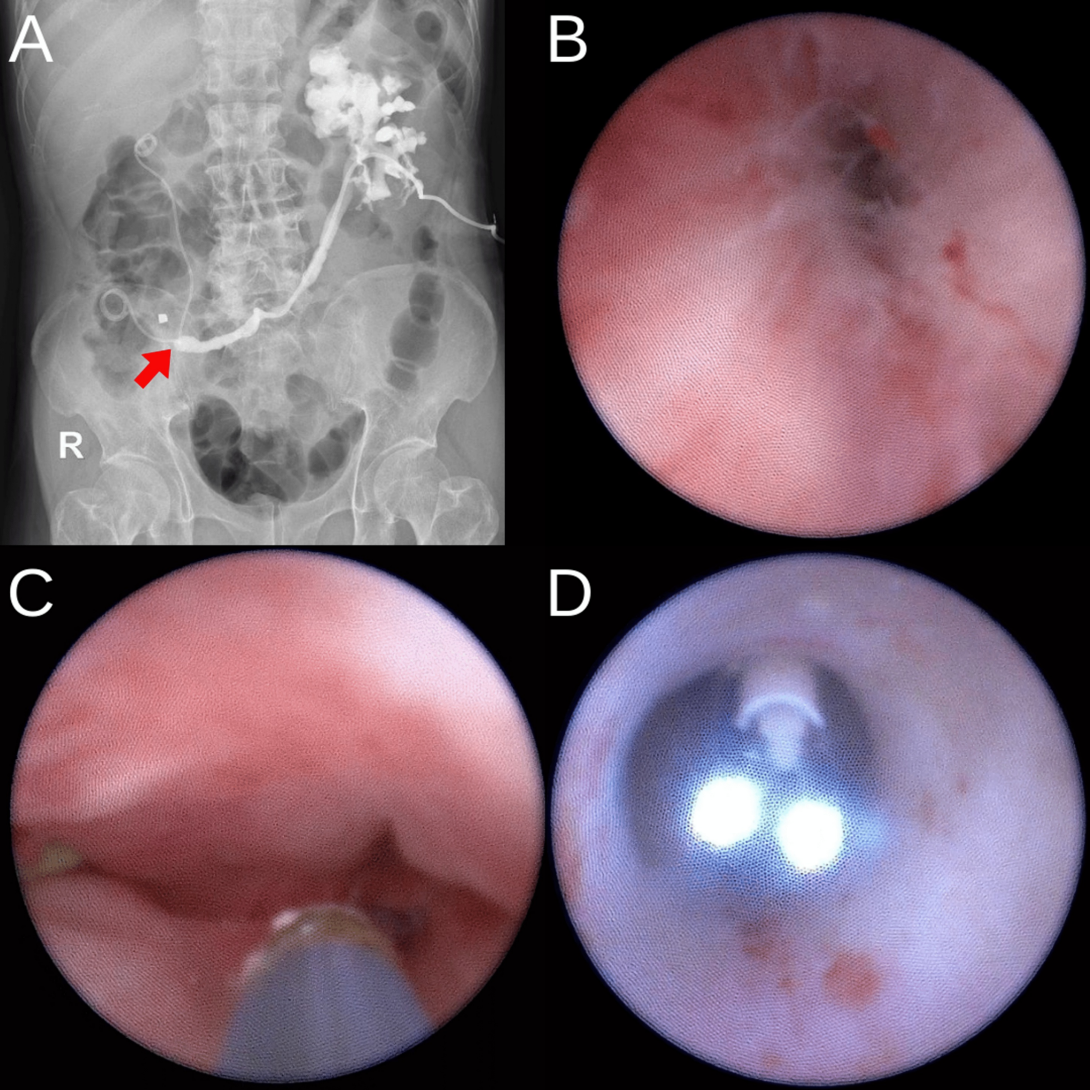
Stenosis of TUUC anastomosis A. Antegrade contrast pyelography showed the stenosis in TUCC (red arrow). B. Stenosis in TUUC anastomosis. C. Antegrade stent in ureter D. Wire to open the tract of TUUC. TUUC: transuretero-ureterocutaneostomy

Eighteen months after surgery, in March 2024, the patient presented with left flank pain and oliguria. Ultrasound and abdominal X-rays showed grade 2 left hydronephrosis and an elevated creatinine level of 1.98 mg/dL. URS through the TUUC orifice revealed total stenosis at the TUUC anastomosis of the left ureter. A left nephrostomy was performed, followed by antegrade pyeloureterography, which confirmed the diagnosis of TUU anastomosis stenosis (Figure 1).

**Figure 2:**
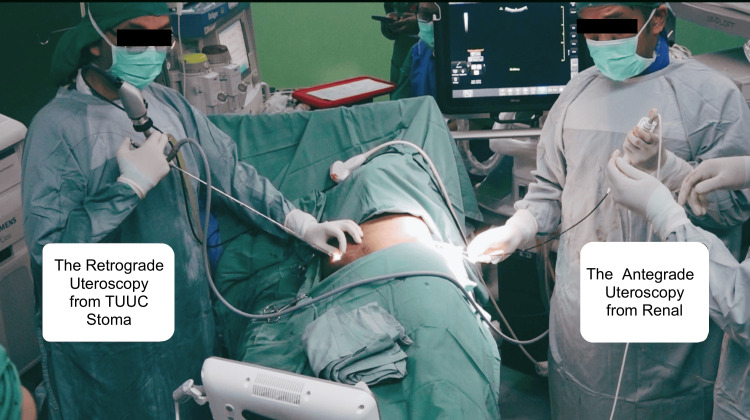
Positioning of the operator in the stenosis TUUC anastomosis surgery TUUC: transuretero-ureterocutaneostomy

The procedure continued with a retrograde 9 Fr semirigid ureteroscope, while the nephrostomy tract was dilated using a 15 Fr mini perc amplatz sheath. Through this sheath, a 7.5 Fr flexible ureteroscope was advanced. The first operator, managing the antegrade approach, accessed the upper ureter via the nephrostomy tract. The second operator, responsible for the retrograde approach, used the 9 Fr ureteroscope to evaluate the anastomosis. After assessing the stricture, the first operator provided light guidance from the antegrade side, while the second operator applied Holmium laser endoureterotomy from the retrograde side. The Holmium laser was set at 2.0 joule, 10 Hz, and short pulses. Finally, the second operator inserted a 6 Fr DJ stent to ensure ureteral patency and proper drainage (Figure 2). A month post-procedure, the patient experienced complete relief of left renal colic, increased urine production through the TUUC (ranging from 1500 to 2200 cc per day), and decreased creatinine levels to 1.36 mg/dL without complications.

## Discussion

Transureteroureterostomy (TUU) is a reconstructive urinary procedure used when the ureter cannot be reimplanted into the bladder. Combined with ureterocutaneostomy (TUUC), TUU involves connecting the ureters across the midline to maintain continuity, typically in patients with a salvageable kidney and unobstructed ureters [2]. However, TUUC is associated with complications, notably anastomotic strictures, which can occur in up to 20% of cases due to ischemia, surgical issues, or pre-existing conditions like radiation-induced fibrosis. Stenosis or stricture can result from ischemia, fibrosis, radiation therapy, or factors like diabetes, intraoperative bleeding, and infections [3]. Ureteral stenting and balloon dilation are commonly used to manage stenosis, with the antegrade approach preferred for its high success rate [4].

Various techniques are used to manage complications, including anastomosis revision, interposition grafts, and minimally invasive methods like balloon dilation or stent placement. Holmium laser endoureterotomy has shown promise in treating benign ureteral strictures, reducing the need for invasive revisions. Antegrade application of the laser is practical for managing strictures after TUUC, with low recurrence rates and minimal thermal damage, promoting better healing and reducing restenosis [5,6]. The Holmium laser endoureterotomy technique is highly effective in enhancing clinical outcomes, with success rates ranging from 79% to 82% for benign strictures, particularly in short, non-ischemic cases. In comparison, balloon dilation shows significantly lower success rates, with only 33-57% success for strictures longer than 2 cm [6].

In our technique, ureteral stenosis resection is done using a Holmium laser with a double approach. This approach allows operation from two angles, ensuring the anastomosis remains intact and precise. It reduces the risk of damage to surrounding tissue and improves the accuracy of resection and anastomosis of the obstructed ureter. This method minimizes the likelihood of scarring or recurrence of strictures post-procedure. The double approach also enables handling more complex stenosis, improving procedural efficiency and reducing the risk of complications or recurrence after the intervention.

This technique's dual approach improves precision, reduces tissue damage, and minimizes recurrence, making it a superior option for managing complex ureteral stenosis. Studies have shown that such combined approaches, which involve both antegrade and retrograde access to the ureter, significantly enhance technical success rates, with one study reporting an 88% technical success rate and 82% stent-free rates at 12 months. This method allows for precise navigation and treatment of ureteral obstructions from two directions, facilitating more effective interventions with lower complication rates [7].

Endoscopic combined intrarenal surgery (ECIRS), a minimally invasive technique combining antegrade nephroscopy and retrograde ureteroscopy, has shown significant efficacy in managing long-segment strictures. ECIRS improves surgical outcomes by offering a dual-access approach, reducing the need for more invasive surgeries and promoting faster recovery with fewer complications. Furthermore, ECIRS has proven particularly effective in high-risk patients, preserving renal function and ensuring better clinical results than traditional open surgeries [8]. Further research can build upon this study to explore its broader applications and potential improvements.

## Conclusions

TUUC is a valuable alternative for urinary diversion after radical cystectomy, although stenosis at the anastomosis site remains a significant challenge. Advances in minimally invasive techniques, particularly antegrade holmium laser endoureterotomy, have shown promise in managing these strictures. This approach allows precise tissue ablation, reducing the risk of restenosis and improving patient outcomes. This case underscores the importance of using advanced, tailored surgical techniques, such as antegrade holmium laser repair, to enhance the effectiveness and safety of TUUC, ultimately improving the quality of life for patients. Acknowledging the need for long-term follow-up and broader studies would enhance the scientific rigour of this work. Future research involving case series or case-control studies is necessary to strengthen the evidence base and improve the generalizability of these findings.

## References

[1] Soerohardjo I, Zulfiqqar A, Yuri P, Hendri AZ (2022). Comparison of ileal conduit and TUUC: a 4 years study. Indones J Urol.

[2] Manjunath DA, Radhakrishna V, Vepakomma D (2021). Transureteroureterostomy in children: a retrospective study. Am J Clin Exp Urol.

[3] Liu Z, Zheng B, Hu Y (2022). The cause analysis of benign uretero-ileal anastomotic stricture after radical cystectomy and urinary diversion. Front Oncol.

[4] van der Meer RW, Weltings S, van Erkel AR, Roshani H, Elzevier HW, van Dijk LC, van Overhagen H (2017). Antegrade ureteral stenting is a good alternative for the retrograde approach. Curr Urol.

[5] Gnessin E, Yossepowitch O, Holland R, Livne PM, Lifshitz DA (2009). Holmium laser endoureterotomy for benign ureteral stricture: a single center experience. J Urol.

[6] Lucas JW, Ghiraldi E, Ellis J, Friedlander JI (2018). Endoscopic management of ureteral strictures: an update. Curr Urol Rep.

[7] Keoghane SR, Deverill SJ, Woodhouse J, Shennoy V, Johnston T, Osborn P (2019). Combined antegrade and retrograde access to difficult ureters: revisiting the rendezvous technique. Urolithiasis.

[8] Aravind S, Jain R P, Palaniyandi V, Sekar H, Krishnamoorthy S (2025). Endoscopic synergy: endoscopic combined intrarenal surgery (ECIRS)-guided "Cut-to-light" holmium laser retrograde endoureterotomy in ureteral stricture management. Cureus.

